# The social determinants of health and health service access: an in depth study in four poor communities in Phnom Penh Cambodia

**DOI:** 10.1186/1475-9276-11-46

**Published:** 2012-08-17

**Authors:** Sann Chan Soeung, John Grundy, Hean Sokhom, Diana Chang Blanc, Rasoka Thor

**Affiliations:** 1 Deputy Director General, Manager National Immunization Program, Ministry of Health, Phnom Penh, Cambodia; 2 Nossal Institute for Global Health, University of Melbourne, Melbourne, Australia; 3 Director Centre of Advanced Studies, Phnom Penh, Cambodia; 4 Regional Adviser Immunization, UNICEF Bangkok, Bangkok, Thailand; 5 UNICEF Phnom Penh, Phnom Penh, Cambodia

**Keywords:** Health inequity, Social determinants, Urban health, Health access, Urban poor

## Abstract

**Background:**

Increasing urbanization and population density, and persisting inequities in health outcomes across socioeconomic groupings have raised concerns internationally regarding the health of the urban poor. These concerns are also evident in Cambodia, which prompted the design of a study to identify and describe the main barriers to access to health services by the poor in the capital city, Phnom Penh.

**Sources and Methods:**

Main sources of data were through a household survey, followed by in-depth qualitative interviews with mothers, local authorities and health centre workers in four very poor communities in Phnom Penh.

**Main findings:**

Despite low incomes and education levels, the study communities have moderate levels of access to services for curative and preventive care. However, qualitative findings demonstrate that households contextualize poor health and health access in terms of their daily living conditions, particularly in relation to environmental conditions and social insecurity. The interactions of low education, poor living conditions and high food costs in the context of low and irregular incomes reinforce a pattern of “living from moment to moment” and results in a cycle of disadvantage and ill health in these communities. There were three main factors that put poor communities at a health disadvantage; these are the everyday living conditions of communities, social and economic inequality and the extent to which a society assesses and acts on inequities in their health care access.

**Conclusions:**

In order to improve access to health and health services for the urban poor, expansion of public health functions and capacities will be required, including building partnerships between health providers, municipal authorities and civil society.

## Background

Globally, it is projected that the current 3.3 billion urban populations is expected to grow to 4.9 billion by 2030, with the most rapid growth occurring in the urbanized areas of Africa and Asia [1]. Squatters constitute about 20 per cent of all households, with these households and communities being characterised by insecurity of tenure, marked inequality, exploitation and social disruption [2].

Analyses of health patterns and health access in modern urban slum communities illuminate similarities across the globe. These include poor outcomes for maternal health, higher rates of teenage pregnancy [3] and financial barriers to child health care in Nairobi [4]; high rates of respiratory conditions in children under the age of five years due to poor environmental conditions in Karachi slums [5]; and lower body mass index amongst the poorest child slum dwellers in Dhaka [6]. Studies and reviews highlight the multi dimensional nature of poverty and ill health in urban slums including the diversity and heterogeneity of social structure and health access [7], and recommend a range of responses ranging from targeted medical interventions, to capacity building and skills transfer, to infrastructure development and improved networking of NGOs, municipal governments and private practitioners [8].

Cambodia is one modern State experiencing rapid economic growth, urbanization and social disruption. Since the devastating Khmer Rouge period between 1975 and 1979 when 1.7 million died (from a population of 6 million) and the cities were emptied of their urban residents to rural labor camps, Cambodia has undertaken a sustained social and economic recovery, with GNI increasing from $280 in 1995 to $430 in 2006 [9]. Cities, stimulated by a flourishing garment industry, service industries and tourist trade are undergoing significant explosion, with the percentage of populations residing in urban areas increasing from 12% in 1990 to 20% in 2006 [10]. According to the most recent census figures (2008), the annual growth rate of the population in Cambodia (total population 13.4 million) is 1.54% (compared to .5% and 1.4% in Thailand and Vietnam respectively), but with an annual urban growth rate of 2.55% [11].

Associated with these patterns of urbanization and economic growth has been the development of urban slums. An assessment of the status and numbers of the poor in 2001 established that there were about 35,000 families (180,000 people) living in low-income settlements within Phnom Penh’s seven municipal districts. According to one more recent report, poverty stands at 12 per cent of the city population. The poorest areas include a total of 22 different recognized squatter settlements, including six in which a health insurance scheme is located [12]. Since 2000, various initiatives have been undertaken to improve the health of those in poor communities, including the establishment of an Urban Health Project and the establishment of health insurance schemes for the poor referred to as “health equity funds [13].”

However, despite these initiatives, significant challenges remain in meeting the needs of the poor. More broadly across Cambodia, despite significant health system developments in recent years, communities in the lowest socio-economic quintiles have substantially less access to health services than their wealthier counterparts, particularly for facility based care [14,15]. The National Immunization Programme (NIP) in 2005 identified up to 16 per cent of communities in Phnom Penh (109 of 695) as at-risk of higher disease transmission of vaccine preventable diseases due to low coverage of services [16]. One of these low immunization coverage communities in the centre of Phnom Penh in 2005 was the source of a vaccine-derived polio case in 2005 and 2006 [17], which prompted national immunization campaigns and other targeted strategies for populations at risk of infection from vaccine-preventable diseases.

## Aims and objectives

The purpose of this study, undertaken collaboratively by the Ministry of Health, Centre for Advanced Studies (an independent Cambodian research agency), and UNICEF was to generate sufficient insight into barriers to health access in order to develop strategies for improving communication with and health access for at-risk populations in Phnom Penh. The specific objectives of the study were as follows:

1. To identify and describe the main barriers to access of health services, through group discussion and interviews with community members, health centre staff and local authorities

2. To identify and describe health system delivery approaches to improve and sustain health service access for maternal and child health care among at-risk populations.

## Research methods

### Data collection

The study adopted a mixed methods approach to gain the needed in-depth understanding of the barriers and potential solutions. The study was conducted in three stages. In the first stage, researchers visited the districts and health centres to identify the poorest populations.

In the second stage, a standardized household questionnaire was used to randomly survey 160 mothers of children younger than five years in the four selected communities. The primary purpose of this household data collection was to gather background information of respondents and their overall knowledge, attitude and practices regarding maternal and child health care services as a prelude to more in depth qualitative interviews. Eight researchers from the Centre for Advanced Studies (CAS) conducted the household survey, with supervision by municipal and national health authorities, a UNICEF consultant and senior CAS researchers. A two-day orientation with the researchers and one-day testing of the questionnaire took place to ensure good-quality data collection.

The third stage of the study consisted of qualitative interviews in the four communities. In depth interviews were conducted with 20 health centre staff and key informants. Four small focus group discussions (FGD) were conducted with health centre teams and four with mothers (2 per community). The FGDs ranged in size from 8 to 20 participants and relied on open-ended questions. A questionnaire guideline was designed for use with the mothers and with local authorities and health centre staff. The FGDs with health centre staff were conducted in the health centre closest to each of the four communities.

### Sample population

The study participants were identified in a step-wise process. The health centres were selected purposefully by Operational Districts themselves as having the highest-risk communities in their catchment area. In the next step, communities were purposefully selected by health centre staff as being the poorest in the catchment area. Community leaders in the four communities were then consulted to identify the poorest areas of that village. As the leaders did not have community lists (or the lists were outdated), and there were inconsistent estimates provided on the numbers of families residing in each area, the researchers decided to divide the targeted poorest village areas into three or four blocks, with 10–15 mothers with children younger than five years selected from each block. For the household data collection, researchers moved from house to house in each block until 40 mothers had been surveyed with the standardized household questionnaire

For the qualitative interviews, the researchers were asked to identify mothers and/or key informants who during the household survey could articulate the social context and barriers to health service that they and their neighbours experience. Thus a majority of the interviewees for FGDs were purposefully selected for the in-depth interviews.

### The study communities

Table 1 outlines the background characteristics as well as the sample sizes for the study in the four communities.

**Table 1:** Study communities Phnom Penh access study

**Community**	**Community type**	**No. of families**	**Sample**
**Household survey**			
Community A	Displaced Community	300–400	40
Community B	Riverside & Cemetery Community	300	40
Community C	Inner city Settlement	2,341	40
Community D	Railway Community	89	40
**Total**			**160 mothers**

It is not apparent that these four communities are homogeneous economically, although without doubt the majority of the populations are very poor. Most of the communities have a visible social hierarchy. In Community A, residents are classified as those whom were ‘longer-term’, having been displaced from an original site by fire, and those whom were “renters.” This is often evident in the way the communities are physically structured. In Community B, the poorest communities live closest to the river banks, with more established families in better housing located further up the river banks. The very poor also resided on a nearby cemetery plot. In Community C*,* some families have been relocated into apartment blocks, in contrast to poorer groups who are still residing in temporary housing. In community D*,* the poorest community members live closest to the railway line, and slightly better off households are situated further from the track.

### Data analysis

Following the collection of data through the household survey in stage two, debriefing meetings were conducted in the CAS office. Responses to the few open-ended questions were re-coded before the data was entered into statistical analysis software (Epiinfo). Following the interviews and focus group discussions in stage three, the researchers recorded the summaries (in Khmer language) into thematic areas. Following the interviews and FGDs, the researchers met to cross reference findings and the implications for recommendations.

The approach to the analysis was based on “grounded theory.” The essence of this theory is that truth is most likely to be approximated through ongoing analysis in the field. Analyzing in the field will also ensure that important contextual information can be retained and if necessary followed up in the field. Ongoing analysis in the field will result in the emergence of analytic frameworks and a more focused and consensual level of enquiry [18,19].

As will be elucidated in the findings and discussion sections of this paper, a framework emerged during the process of this study which reflected the social determinants of health (see discussion section). As will be seen, findings are consistent with the observation that there are three main factors that shape patterns of health and illness in communities: these are the everyday living conditions of communities, the distribution of power and resources between social groups and the extent to which a society measures and takes action on inequities in health care access [20,21].

### Study scope and ethical clearance

The research is not a population-based survey with a sampling methodology that produces generalised results regarding health status, knowledge or behaviours which are representative of at-risk populations in Phnom Penh. Rather, the findings provide a detailed analysis of four communities in terms of how community members, health staff and local authorities perceive access to health services for the poor and their opinions on how to improve their access to these services. Further, the research is not a quality assessment of health service provision, although it seeks to understand community members’ perceptions of the quality of the service available to them.

Following explanation of the study objectives, verbal consent was sought from all participants prior to interview. A study proposal was submitted to and approved by the National Ethics Committee of the Ministry of Health in December 2008.

## Results

Results are here separated into the quantitative and qualitative components. The discussion will analyse the main themes that emerged from both sections of the study.

### Quantitative results

#### Socio-economic backgrounds

Of the 160 survey respondents (women with a child younger than five years), the mean age was 29 years and the mean size of household was 5.8 persons. Eighty-four per cent of the respondents were of Khmer ethnicity. Seventy-one per cent of the respondents identified themselves as “migrants”, and 29 per cent identified themselves as “mobile.” On average, the respondents have lived in their community for 7.6 years. Thirty-eight per cent of them moved to their current location from another area in Phnom Penh; 55 per cent came from other provinces. Key professions of the main income earners in each respondent’s family are construction (22 per cent), home or market selling (19 per cent) and motorcycle taxi driving (17 per cent). Other work includes carpentry and electrical repair (11 per cent), secretarial (8 per cent) and government jobs (7 per cent).

Only 57.5 per cent of the respondents had completed primary education. Given the low education levels, it is not surprising that the income levels in the families surveyed are also low. Sixty-two per cent of the respondents stated that the household income is between $1.25 and $5 per day. This contrasts with the last estimate of the Phnom Penh Municipal Government of the GDP per capita for Phnom Penh City was reported to be 820 USD in 2005 [22]. In the three months prior to the survey, the mean expenditure by households on health care was $66. Twenty-five per cent of the respondents’ households spent $100 or more.

Exacerbating the issue of high health care costs is the fact that only 14 per cent had a ‘poverty card’ or ‘insurance card’, which exempted them from fees for certain health care services.

In summary, income and education levels are low, and relative to income, health care costs are high with very limited levels of social protection.

#### Health service coverage and use

Immunization coverage in the poor communities is satisfactory. The third dose of diphtheria, pertussis and tetanus, and the hepatitis B vaccines was verified by immunization cards for 88 children of an eligible population of 139 (63 per cent). A further 41 children (29.5 per cent) had been vaccinated, according to the oral history from the mothers. Only 10 of 139 children (7 per cent) were reported by the mothers as not having been vaccinated. Sixty-one per cent of respondents had three or more antenatal care visits for their previous pregnancy. The majority of the mothers stated they received most of the recommended antenatal care services (tetanus vaccination, iron supplementation, advice on nutrition and danger signs). The vast majority of previous deliveries were facility-based, with the majority taking place in public hospitals (48 per cent) and health centres (31 per cent).

The private sector is the first choice for child curative care (50.3 per cent indicated this preference for the last child illness) and health centres/government hospitals are the first choice for preventive care (79 per cent reported the child received the last immunization at a government facility, and 66 per cent indicated that the last reproductive health consultation was received at government facilities). The primary reason provided by respondents for the selection of the provider for the last childhood illness was perceived quality of service (refer to section on qualitative findings for description of “quality”).

Overall, the household findings demonstrate reasonable coverage for health service access as measured by immunization and maternal health, with the private sector the preferred option for illness consultation, and the public sector for prevention services.

### Qualitative study results

Results are organized according to the main themes that emerged from the FGDs. Table 2 summarizes the main qualitative research findings by theme area.

**Table 2:** Summary of qualitative findings and policy and practice implications

**Theme area**	**Detailed finding**
**Social Structure**	Poor communities are complex in structure and do not rely solely on the administrative leadership for social cohesion or social action. Community members often identify more closely with community subgroups, community leaders, NGOs and even resident health private practitioners, and are primarily reliant on their own family and neighbours for assistance. This supports a case for a health promotion strategy to work locally with community subgroups and families and their networks rather than relying solely on the administrative organization and procedures.
**Social Insecurity**	There are many aspects of social insecurity in communities that impact on health and well-being. These include physical, income and health insecurity. This social context for health and well-being indicates that the primary determinants of poor health in these communities can best be understood in structural rather than behavioural terms. This supports a case for a more comprehensive social policy approach to address the structural factors rather than a reliance on health education strategies for individual behaviour change.
**Social isolation**	There are particular subgroups of the poorest families in the four communities that are particularly at high risk of social exclusion and social isolation – these include single mothers, young school-age children (but not attending school) and teenagers. Social programmes should target these most vulnerable groups to provide them with a minimum level of social opportunity for development and social protection.
**Social Protection**	Health workers assess the poverty status of their patients, and patients know they are being assessed for their capacity to pay. As a result, mistrustful relationships can develop between government health centre staff and community members. On the other hand, those people with exemption cards expressed confidence in attending health facilities. This makes the case for extending the health equity fund or related health protection schemes to increase the use of health care services by the very poor.
**Health Networks**	Informal networks are likely to be the most influential factor in determining health care-seeking behaviour. The quality and cost of health care services are routinely discussed among families, friends and neighbours. This being the case, the most powerful advertisement for improving health care and health care access is the quality, attitude and cost of services provided directly to the communities, enabling community members then to share this information through their local social networks.
**Health Markets**	There is no single unified health care system in the urban context. There is instead a health care market with a wide range of choice of provider and type of service, even for the urban poor. The poor are “shopping for health.” A better understanding of the dynamics of this health care market for the poor could guide policy makers towards improving mechanisms for quality health care and social protection.

#### Health and social insecurity among the very poor

Many of the focus group participants expressed feelings of insecurity, which very much relates to their social context rather than individual behavioural constraints. *Physical insecurity* was expressed in terms of night-time disturbances, assaults and abuse of alcohol and drugs. But it was social and income insecurity that was the most predominant theme in the discussion of social context.

*Social insecurity* was expressed in terms of insecurity of land tenure. “We don’t know what will happen to us” and “we don’t know when we will have to move” were common statements from community members in two of the communities.

*Income insecurity* was often expressed in terms of irregularity of income of the main earners in households. Motorcycle taxi drivers, construction workers, hairdressers and markets sellers are all subject to the vagaries of the market place. For most income earners in society, variation in income can be managed through savings or borrowings. But for income earners of US$1–$2 per day, their family lives in a chronic state of insecurity – uncertain of the income that will come, especially for daily nutrition and education needs for children. This is especially the case when income is irregular.

The researchers found that in many cases, the income insecurity led to restrictions on food purchases and indications of under-nutrition. Notably, families will borrow or sell household items when they need to pay for health services, but the daily education costs are often deemed non-affordable. There were frequent reports of children dropping out from school or attending irregularly due to lack of family income.

Surprisingly, *health insecurity* was s not often expressed in terms of the inability to afford health care services. Rather, health insecurity predominantly referred to the poor access to water and sanitation, with the absence of any institutional mechanisms for waste management being a key preoccupation. Community members, local authorities and health workers consistently identified poor waste management, water supply and sanitation as the main threats to the health of families. Most childhood illnesses and even adult illnesses were attributed to uncleared rubbish, lack of toilets, standing water and mosquitoes. Sometimes the problems were attributed to personal and household behaviour, but more often, they were identified as community characteristics that people – even the local authorities – felt powerless to resolve. “The words of the poor are cheap,” explained one long-term resident.

Given these conditions, it is hardly surprising that there is a heightened sense of ‘living for the moment’. It is difficult to undertake or envision long-term community or household planning in this chronic state of daily insecurity and powerlessness. Frequently, the researchers heard community members say they are “living for the day”. One local authority member indicated that many community members do not even live for the day but live from *“moment to moment”* in order to cope with each day’s needs.

#### Social exclusion and isolation

Participants in both the in-depth interviews and focus group discussions talked of exclusion and social isolation, mostly related to the structural determinants of income capacity, education access and powerlessness previously noted.

*Single mothers* in particular are at high risk of exclusion due to absolute income poverty. In one case, a single mother was completely dependent on her neighbours for income and social contact. Because they dropped out of school, many *young adults* are exposed to risks of drug abuse and prostitution.

The researchers found limited examples of community activities or structured gathering locations for young people. In one community, an NGO was active in providing therapy for injecting-drug users, and in other communities, home care visits were conducted by an NGO supporting people who are HIV-positive. Overall, structured services or social activities are not in place for young people in the four communities.

The process of social exclusion starts very early. Repeatedly, community members highlighted the daily income demands of education as a major strain on family income and on social participation. In some cases, NGOs provide education programmes for young children within the community. In other cases, NGOs provide income support for children to attend schools. Local authorities try to help the children of poor families through the provision of a letter to the school teacher exempting them from paying school fees (as is the case with certain health care services).

The depth of social exclusion is perhaps expressed most clearly in Dam Slaeng where makeshift homes crowd around burial plots in the cemetery and children run between the tombstones. “The children are not afraid of the ghosts – the ghosts are afraid of the children,” one resident commented. In one abandoned building, six families had set up blanket partitions to serve as makeshift walls to separate sleeping areas. Some of the current residents in the community had only recently moved there due to newly impoverished circumstances. One woman said she had lived there since the early 1980s.

#### Social vulnerability and protection

The social vulnerability of the urban poor in these communities was expressed in both behavioural and structural terms. In terms of behavioural expression of vulnerability, community members reported that they are “looked at” by health staff to determine whether they had the capacity to pay, in order to decide who is treated first. “You have to have money. If you do not have money, they won’t pay much attention to us,” explained one resident. In all four communities, health centre staff indicated that they exempt the very poor from payment for certain health services. However, those health workers also indicated that in the absence of a poverty card or a letter of exemption from the local authority, they will look at the clothing or personal items of a patient to make an on-the-spot poverty assessment.

The absence of systematic social protection mechanisms increases the risk of a mistrustful relationship between health professionals and clients. This equally applies to the relationship between the education sector and community, with some people indicating that children are “afraid” of the teacher if they do not have enough money to pay for school expenses.

There were many examples of vulnerability determined by structural factors. The daily struggle to manage family food, education and health care costs with a low income was a consistent theme spoken of throughout this research.

According to one local authority official, “So if we think about it, health and education and food, they spend more on education – they have to spend on education every day…when they do go to school they often stop at level two or three…they just don’t have the capacity to send them to school.”

And one mother commented, “I have two children going to school, but one has had to stop…because we have no money for the teacher. Our family is spending more money than our income. We have no rice field or garden. For health care, we pay money every now and then, but for education you have to pay every day and for food we have to spend most of all.”

Families use various coping mechanisms for their day-to-day survival and basic needs. For health care, they typically sell household or personal property, borrow from a family member or neighbours, seek out NGO or pagoda support, or ask for assistance from the local authority (letter of poverty status to exempt them from certain fees). Health centre staff indicated that they do not ask the poorest of the poor to pay, but there were many cases in which people did not seek out health care, opting for exclusion or social restriction.

#### Demand and supply of health care services

Perception of quality of care was the main determinant from a client perspective for selection of provider (refer also to quantitative findings). From the community perspective, quality was often defined in terms of hygiene or technology, such as “the hospital is very clean” or “they have all the modern equipment”, or in terms of outcomes, such as “the medicine is very effective” or “the child gets better quickly”. The community members often cited the perceived skill of the provider as being critical when they were seeking health care. On the other hand, a provider with a poor attitude is viewed very dimly by clients. The poor attitude was interpreted mostly in terms of waiting longer because you are poor, being looked at judgmentally to see if you are poor or not, and impolite speech. All of these quality factors seem to influence people’s selection of provider.

What was apparent from the provider perspective was that, since the closure of outreach services in 2007 and the switch to a “fixed facility” approach (where services are only provided at the health centre and not in the community), there has become less clarity as to where the unreached populations are situated and what needs to be done to reach them. When marking hard-to-reach or slum areas on health centre catchment maps, the health centre workers demonstrated knowledge in locating them but they expressed less confidence in identifying pockets of non-immunized children. Comments, such as the following, indicated the health centre staff’s uncertainty of population coverage in high-risk areas:

"“We are not sure what is going on there now.”"

"“Funding for outreach has stopped so we cannot be sure where they are.”"

"“These places are confusing – people are coming and going all the time.”"

It was also not clear whether social mobilization and communication meetings were taking place regularly enough with local authorities and village volunteers. Even though a fixed-facility site strategy relies on population demand, funding is still required for health education and social mobilization in communities for the fixed facility strategy to work. Yet when asked to define their function in relation to health, local authorities saw their role more in terms of gathering statistics and social mobilization and less so in actually mobilizing resources for public health interventions.

In summary, as previously noted, although community members, health workers and local authorities consistently pinpointed social and economic conditions as the prime determinants of poor health, there was little evidence that health service systems are oriented to public health or on taking actions on the social determinants of health.

## Discussion and conclusions

### Strengths and limitations of the approach

One of the main limitations of this study was the inability to define a “target population” with a specific population denominator that would enable statistically relevant measures of health care coverage for the urban poor. As outlined in the literature, urban poor communities are by no means homogeneous in terms of economic and social make up. It was therefore difficult to determine a specific boundary to these communities, which would enable classification of a population as “very poor.” The indistinct nature of urban poor geographic and social boundaries therefore required a switch in methods to purposeful selection of high risk communities (as described above) as well as of purposeful selection of participants for qualitative studies. The other implication was that household surveys were applied for the primary purpose of painting a contextual picture which the background for the more in depth information generated through in depth interviews and FGDs. This lack of statistical relevance may therefore call into question the generalizability of findings on health care access.

**Figure 1:**
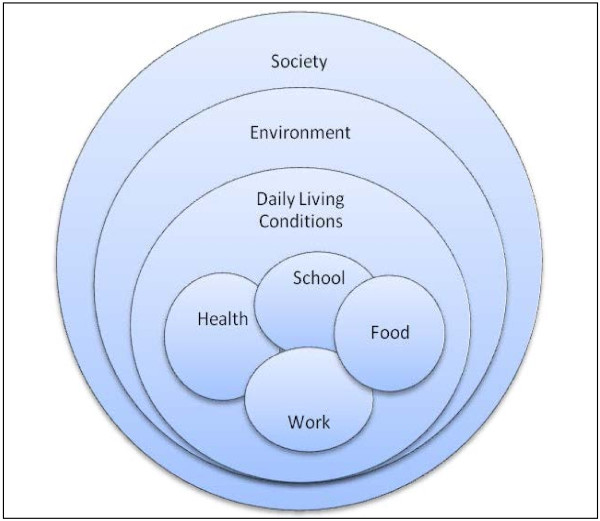
Framework for analysis of the social determinants of health.

On the other hand, complementing rapid surveys with qualitative studies in urban poor settings deepens understanding of the determinants of poor health care access. The sourcing of information from literature, household surveys and in depth interviews assisted in triangulating data from multiple sources. The development of an analytic framework by the research team at the mid way point of the study enabled to the researchers to build up an overall picture of the social determinants of health (see Figure 1). We would argue therefore that triangulation of data sources, and the use of these sources to reach a research team consensus on an analytic framework was an important factor contributing to the validity of the study findings.

It is in this sense that the methodological approach adopted in this study may have wider applications for responding to the health needs of the urban poor. Through purposeful selection of high risk communities in an urban setting, combined with rapid household assessment of health access, further complemented by in depth discussion of the determinants of poor access with these communities, it is possible to construct an overall picture of health needs and the related policy and planning actions required to respond to these needs. This has also been the experience in the Mongolian urban health setting, where integration of rapid household assessments combined with more in depth qualitative evaluation in high risk areas in Ulaanbaatar has triggered the national scale up of a health planning strategy for both the urban and rural poor [23].

### Theoretical frameworks for understanding access to health and health services for the very poor

In this study, the sense of “entrapment” by respondents inside a poverty setting was not only evidenced by the average length of stay of respondents in these communities, but also by the repeated assertion that the principal source of poor health outcomes are the unhealthy social and environmental conditions in which people live on a daily basis. The combination of barriers in access to education services, low incomes and poor environmental conditions means that families are trapped in poor living environments which put them at chronic risk of communicable disease. This being the case, communities asserted that it was the social and environmental conditions rather than access to medical services that are the main barriers to sustaining and improving family and community health. As Figure 1 demonstrates, access to health services is viewed as just one “slice” in an overall pattern of daily living that reinforces exclusion and distance from social engagement and opportunity.

As Figure 1 demonstrates, limited work opportunity means that people cannot always afford to meet the recurrent expenses of food, health and education. Children without a basic education then find it difficult when they are young adults to escape this cycle of poverty and deprivation. People live in an environment they report negatively affects their health, resulting in high health care expenditures and poor health. Families then become “locked” into a system of poverty which distances them from wider society both functionally and spatially. Solutions therefore require structural or ‘systematic’ approaches. Health and health access are therefore not a distinct sector or “slice” of life. It is meshed with the reality of daily living conditions with its backdrop of surrounding environmental space and limited access to various forms of social support.

This “social distancing” of the poor from mainstream society has also been noted in historical context, whereby advances in industry and technology has made it increasingly possible for the more advantaged to separate themselves spatially from those in conditions of disadvantage [2]. This trend of growing spatial segregation is matched by social and economic segregation that the poverty trap in Figure 1 describes.

These findings and the associated analytic framework are consistent with recent policy and practice developments in relation to the social determinants of health [24,25] and primary health care [26]. This paradigm shift reorients health service delivery from a focus on provision of essential medical service packages to include a wider health policy and practice framework which includes engagement with other sectors and community stakeholders including municipal authorities and NGOs. The results of this Cambodia study are also consistent with the findings from studies across the world as described in the introduction to this paper, demonstrating the multi dimensional character of poverty and the impact this has on health and health access for slum dwellers.

The demographic trends in Asia of increased urbanization, high rates of population mobility and persisting income inequalities have raised concerns regarding the effectiveness of current social policies and health system design. That is, current thinking is likely inadequately addressing inequities in health access and health outcomes, not only between urban and rural populations, but more importantly between high and lower socio-economic groupings within these geographic settings. Current social theory indicates that health outcomes and access are attributable to a chain of environmental, social and behavioural events that operate from the social macro-level down to the level of the households [27]. The poorest households, as demonstrated by this study, often experience social isolation, as measured by low levels of social engagement in the economy and social sectors (education and health) and the corresponding low level of “instrumental supports” provided through mainstream state institutions, municipal authorities and civil agencies.

But what actions can a health sector undertake to address the issues of social isolation and poor health in urban areas?

### The health and social policy response

The recommendations from this study (see Table 3) highlights the need to establish a better balance in health system design and delivery between the requirement for higher quality and affordable primary medical care and the need to focus on primary prevention through strengthening of community and municipal level health partnership networks.

**Table 3:** Recommendations health access study Phnom Penh

**Recommendation subject area**	**Detail**
1. Resourcing Communication	Adequate resources for health centres are needed for health education and services outreach to at-risk communities. The additional resources would i) strengthen links between health services, community practitioners, local authorities, NGOs and communities, ii) establish contact with and support local social networks for health (formal and informal) and iii) provide mobile services for the most at-risk populations.
2. Improving Service Quality	A combined health education and quality improvement strategy should be adopted so that poor families can access better quality and more affordable care for sick children from health centres (for example, facility and community IMCI).
3. Focussing On Health Monitoring	The Municipal Health Department (MHD) needs to undertake a systematic approach to surveillance of at-risk populations through the support of district health centres. In conjunction with local authorities and civil society partners, the MHD should conduct regular mapping and micro-planning for at-risk populations. Such mapping and micro-planning should be built into the routine functioning of the surveillance and planning system so that surveillance focuses both on disease and on detecting health risks and health inequities, specifically for childhood immunization, primary school retention, health insurance status, anthropometric assessment/food security measures and environmental health.
4. Building Public Health Function	A review of essential public health functions for urban health should identify resources required; a capacity-building plan is needed to strengthen the delivery of essential public health functions, either through local authorities, NGOs, health centres or a combination of all (waste management, nutrition surveillance, health monitoring etc).
5. Expanding Social Protection	Social safety-net equity funds, based on a model of the health equity fund, need to be established in the poorest communities in Phnom Penh on a comprehensive basis to ensure access to health care and education services for the very poor.

Current paradigms of monitoring, as exemplified by the “disease surveillance” paradigm of communicable disease control, are in some ways symptomatic of the delayed response of the health system to social transition. The disease focus of monitoring, as exemplified by the “disease outbreak response” approach, is of course efficacious in control of disease outbreak and spread. However, as exemplified by the outbreak of vaccine derived polio virus in very low coverage areas in Phnom Penh in 2005, it is the poor-quality active surveillance in areas of low coverage (and associated pre emptive health action) that in the end exposes these communities to high levels of communicable disease risk. That is, although systems are designed for disease detection and outbreak response, communication and social systems are not oriented towards early detection of low coverage areas and subsequent prompt follow up interventions. These findings, and the recommendations on surveillance linked to these findings, are supported by several studies which demonstrated that the specific characteristics of urban health settings requires detailed mapping and assessment of health needs so that context specific responses to the problem can be designed and implemented [20,28].

This imbalance between health and disease perspectives in health planning is also evident in the lack of clearly delineated public health functions for either health or municipal authorities, particularly with regards to waste management, safe water, youth affairs and substance abuse programs. This finding is consistent with a review of the role of municipal health authorities globally in reduction of health inequities, which indicated that only 17% of authorities had a defined role in this regard [29]. This is of particular concern given the demographic trends alluded to above, as well as the generalized patterns of urbanization and decentralization that are emerging in free market societies in Asia and elsewhere.

Clearly, these two major issues of balancing of strategy – that is of health and disease surveillance on the one hand, and development of public health function with provision of medical services on the other, are significant longer term health and administrative system developments that will be required to reduce health inequities. In the shorter to medium term, as the recommendations in Table 3 describe, scaling up of health protection, improvements to service quality and where applicable, community-based service delivery and health education programs are practical steps that could be undertaken with moderate resource commitments.

However, for longer term and sustainable development, leadership or effective “stewardship” [30,31] of the sector will be required in order to commit health system managers and workers to social accountability for reaching out to the urban poor. Sole reliance on contractual and fee for service models of management for health centres using fixed facility strategies is likely to be in many cases isolating for the very poor in terms of their capacity to afford and access quality of care, and furthermore, to make any significant changes to the determinants of their daily conditions of life. From the provider side, adequate health surveillance and social protection measures will need to be instituted so that these populations are detected, their needs assessed and programs subsequently designed and financed to reach out to them.

As various analysts have outlined, in emerging free market societies, very careful management is required of the central and decentralized functions of the state, in order to negotiate the complex and systematic challenges of social disruption linked to rapid economic growth and rapidly urbanizing societies [2,32]. The vacuum in management that has resulted from the decline of the command and control state models needs to be filled by cooperative social and institutional networks, that international reviews and studies are now beginning to describe as being essential for addressing persisting issues of inequities in health, particularly for slum dwellers [8,33]. In concordance with global level policy developments in relation to the social determinants of health and primary health care, more direct actions will be required by health policy makers to expand the public health functions and capacities and partnerships of health providers, municipal authorities and civil society in order to improve the daily environmental and social conditions of life, the absence of which community members in this study identify as being the main source of their poor health.

## Competing interests

The authors declare they have no competing interests.

## Authors’ contributions

SC provided overall strategic direction and oversight for the research. JG drafted the design and ethics submission, conducted the literature review, participated in data collection and drafted the initial paper. S led the local research team and revised data collection instruments. He also participated in the field research, oversaw analysis and reviewed report drafts. DCB and RT provided technical inputs into design and substantially revised manuscript drafts. All authors read and approved the final manuscript.

## References

[B1] United Nations Population Fund Unleashing the Potential of Urban Growth UNFPA. State of World Population 2007 2007 New York: UNFPA

[B2] UN Habitat The Challenge of Slums Global Report on Human Settlements 2003 http://www.unhabitat.org/downloads/docs/GRHS.2003.2.pdf

[B3] Taffa N A comparison of pregnancy and child health outcomes between teenage and adult mothers in the slums of Nairobi, Kenya Int J Adolesc Med Health 2003 15 4 321 329 14719414 10.1515/ijamh.2003.15.4.321

[B4] Taffa N Chepngeno G Determinants of health care seeking for childhood illnesses in Nairobi slums Trop Med Int Health 2005 Mar 10 3 240 245 10.1111/j.1365-3156.2004.01381.x 15730508

[B5] D'Souza RM Housing and environmental factors and their effects on the health of children in the slums of Karachi, Pakistan J Biosoc Sci 1997 29 3 271 281 10.1017/S002193209700271X 9881135

[B6] Pryer JA Rogers S Normand C Rahman A Livelihoods, nutrition and health in Dhaka slums Public Health Nutr 2002 5 5 613 618 12372153 10.1079/PHN2002335

[B7] Agarwal S Taneja S All slums are not equal: child health conditions among the urban poor Indian Pediatr 2005 42 3 233 244 15817971

[B8] Sheuya SA Improving the health and lives of people living in slums Ann N Y Acad Sci 2008 1136 298 306 Epub 2007 Oct 22 10.1196/annals.1425.003 17954669

[B9] The World Bank The Little Green Data Book 2007 http://siteresources.worldbank.org/ESSDNETWORK/Resources/LGDB2006.pdf [accessed on line July 30 2008]

[B10] UNESCAP Rate of Urbanization [Accessed on the web July 30 2008] http://www.unescap.org/Stat/data/syb2007/2.1.Urbanization.xls

[B11] National Institute of Statistics Cambodia General Population Census of Cambodia 2008 NIS Phnom Penh: Ministry of Planning Phnom Penh

[B12] Dr Peter Leslie Annear (RMIT University) Study of financial access to health services for the poor in Cambodia Phase 2 In-depth analysis of selected case studies Dr Peter Leslie Annear (RMIT University) 2007

[B13] Bigdeli M Annear P Barriers to Access and the Purchasing Function of Health equity Funds Bulletin WHO 2009 87 560 564 10.2471/BLT.08.053058 PMC2704035 19649372

[B14] Ministry of Planning Cambodia Demographic Health Survey 2005 Ministry of Planning www.measuredhs.com

[B15] Grundy J Khut QY O S Annear P Ky V Health system strengthening in Cambodia—A case study of health policy response to social transition Health Policy 92 2009 107 115 19501425 10.1016/j.healthpol.2009.05.001

[B16] Data sourced from National Immunization Program 2008 Cambodia: National Immunization Program, Ministry of Health Phnom Penh

[B17] Okonko IO Ogun AA Adedeji AO Akanbi OA Udeze AO Motayo OB Circulating vaccine-derived poliovirus and its implications for polio surveillance and eradication in Nigeria: A review of the literature Sci Res Essay 2009 4 5 398 418

[B18] Helene S Susan Brown T Choose Your Method: A Comparison of Phenomenology, Discourse Analysis, and Grounded Theory Qual Health Res 2007 17 1372 10.1177/1049732307307031 18000076

[B19] Kevin D A health researcher’s guide to qualitative methodologies Aust N Z J Public Health 2007 31 433 437 10.1111/j.1753-6405.2007.00114.x 17931290

[B20] Abbas B Hanifi SMA Shehrin Shaila M Action Monitoring for Equity and Gender in Health J Health Pop Nutr 2008 26 3 378 383 18831232 10.3329/jhpn.v26i3.1902 PMC2740709

[B21] Navarro V Whitehead M Doran T Burstrom B Helmert U Costa G Navarro V Summary and conclusions of the study The political and social contexts of health 2004 Amityville, NY: Baywood Publishing Company 221 226

[B22] Phnom Penh Municipal Government The http://www.phnompenh.gov.kh/phnom-penh-city-facts-99.html [accessed July 23rd 2012]

[B23] Lhamsuren K Choijiljav T Budbazar E Vanchinkhuu S Chang Blanc D Grundy J Taking action on the social determinants of health: improving health access for the urban poor in Mongolia Int J Equity Health 2012 11 15 (20 March 2012) http://www.equityhealthj.com/content/pdf/1475-9276-11-15.pdf 10.1186/1475-9276-11-15 22429615 PMC3349495

[B24] Marmot M Friel S Bell R Tanja A Houweling J Taylor S on behalf of the Commission on Social Determinants of Health Closing the gap in a generation: health equity through action on the social determinants of health Lancet 2008 372 1661 1669 10.1016/S0140-6736(08)61690-6 18994664

[B25] Disparities PBH Equity H Concepts and MeasurementAnnu Rev Public Health 2006 27 167 194 10.1146/annurev.publhealth.27.021405.102103 16533114

[B26] WHO World Health Report 2008 Primary Health care 2008 Geneva: WHO

[B27] Berkman S Glass T Brisette I Seeman T From Social Integration to health: Durkheim in the new millennium Soc Sci Med 2000 51 843 857 10.1016/S0277-9536(00)00065-4 10972429

[B28] Agarwal S Taneja S All slums are not equal: child health conditions among the urban poor Indian Pediatr 2005 42 3 233 244 15817971

[B29] Patricia AC Michael VH The role of urban municipal governments in reducing health inequities: A meta-narrative mapping analysis Int J Equity Health 2010 9 13 25 May 2010 10.1186/1475-9276-9-13 20500850 PMC2893183

[B30] Ferroussier-Davis O Saltman RB The concept of stewardship in health policy Bulletin WHO 2000 78 6 PMC2560782 10916910

[B31] WHO World Health Report 2000 2000 Geneva: WHO

[B32] Szreter S Rapid Economic Growth and the “4 D’s” of disruption, deprivation, disease and death: public health lessons from nineteenth century Britain for twenty first century China? Trop Med Int Health 1999 4 2 146 152 10.1046/j.1365-3156.1999.00369.x 10206269

[B33] Sclar ED Garau P Carolini G The 21st century health challenge of slums and cities Lancet 2005 365 9462 901 3 10.1016/S0140-6736(05)71049-7 15752535

